# Comparison of the molecular FluoroType Mycobacteria VER 1.0 and the Maldi BioTyper Mycobacteria assays for the identification of non-tuberculous mycobacteria

**DOI:** 10.1128/jcm.01206-24

**Published:** 2024-12-11

**Authors:** Jennifer Guiraud, Caroline Piau, Cécilia Enault, Emilie Nkpa Charron, Danièle Ducos, Christine Lafuente, Armelle Ménard, Olivia Peuchant

**Affiliations:** 1 Laboratoire de Bactériologie, CHU Bordeaux 36836 , Bordeaux, France; 2 Univ. Bordeaux, Centre national de la recherche scientifique (CNRS), UMR 5234 Fundamental Microbiology and Pathogenicity 131819 , Bordeaux, France; 3 Laboratoire de Bactériologie, CHU Rennes 36684 , Rennes, France; 4 Groupe Hospitalier Universitaire Carémeau, Service de Microbiologie et hygiène hospitalière, Nîmes, France; 5 Univ. Bordeaux, INSERM UMR1312, BoRdeaux Institute of onCology BRIC 640680 , Bordeaux, France; The University of North Carolina at Chapel Hill School of Medicine, Chapel Hill, North Carolina, USA

**Keywords:** nontuberculous mycobacteria, identification, diagnosis, MALDI-TOF MS, FluoroType

## Abstract

Accurate identification of non-tuberculous mycobacterial (NTM) species is crucial for the diagnosis and appropriate management of NTM infections. This study aimed to evaluate the performance of two assays, FluoroType Mycobacteria VER 1.0 and Maldi BioTyper (MBT) Mycobacteria. The two assays were evaluated using 119 NTM, including 85 slow-growing mycobacteria and 34 rapid-growing mycobacteria, representing a total of 33 species isolated in three French clinical laboratories. We used the GenoType assays as reference method for species identification, followed by 16S rRNA gene sequencing if the GenoType kits returned *Mycobacterium* sp. Compared to the reference method, the FluoroType Mycobacteria assay provided correct species identification in 89.9% of cases (107/119). Among the most frequently encountered species in clinical settings, low concordance was obtained for *Mycobacterium intracellulare* (82.4%, 14/17), *Mycobacterium gordonae* (66.7%, 6/9), and *Mycobacterium xenopi* (75%, 6/8). Misidentification was obtained in two cases (*Mycobacterium smegmatis* instead of *Mycobacterium mageritense*, and *Mycobacterium mucogenicum* instead of *Mycobacterium phocaicum*). Using the MBT Mycobacteria assay, 78.1% (93/119) of NTM isolates were correctly identified at the species level. One *Mycobacterium europaeum* isolate was misidentified as *M. intracellulare*/*Mycobacterium chimaera*. In five cases, the assay provided more accurate NTM identification compared to GenoType assays, in which closely related species are identified as a group. The FluoroType Mycobacteria VER 1.0 and the MBT Mycobacteria assays are useful tools for NTM identification from positive cultures, reducing handling time compared to GenoType assays. Their routine use in laboratories must take into consideration their performance and limitations in clinical settings.

## INTRODUCTION

Non-tuberculous mycobacteria (NTM) can cause both pulmonary and extrapulmonary diseases, with the skin, soft tissues, and lymph nodes being the most frequently affected (1). Given their environmental presence, accurate identification of NTM is crucial for assessing clinical relevance and managing suspected mycobacterial diseases. Antibiotic regimens and treatment outcomes vary significantly among species and subspecies (2, 3). Yet, a definitive gold standard method for NTM species identification remains elusive.

Identification of NTM isolates can be performed using molecular methods, such as gene sequencing (16S rRNA gene, *hsp65, rpoB*, and internal transcribed spacer) (4). Commercial line probe assays, for example, the GenoType Mycobacterium CM/AS/NTM-DR assays (Hain Lifescience GmbH, Nehren, Germany), are also widely used due to their reliability and ease of implementation and interpretation (5–10). These three commercial kits allow the identification of a total of 31 clinically relevant NTMs and the molecular detection of resistance to macrolides and aminoglycosides in *Mycobacterium chelonae*, in members of the *Mycobacterium avium* complex and *Mycobacterium abscessus* species.

Recently, a new molecular assay, the FluoroType Mycobacteria VER 1.0 (Hain Lifescience GmbH, Nehren, Germany), has been developed. Based on the LiquidArray technology, this assay combines multiplex PCR with asymmetrical PCR and melting curve analysis. It enables the differentiation of 32 clinically relevant NTM species, including the subspecies *M. abscessus* subsp *abscessus*, *M. abscessus* subsp *bolletii*, and *M. abscessus* subsp *massiliense*, from a positive culture.

Additionally, matrix-assisted laser desorption/ionization time of flight mass spectrometry (MALDI-TOF MS) is increasingly being used for NTM identification in laboratories. Updated databases and optimized protein extraction procedures have enhanced its suitability for diagnosis (11–13). For instance, the Maldi BioTyper (MBT) Mycobacteria Library v6.0 (Bruker Daltonics, Bremen, Germany) currently includes spectra of 182 mycobacteria species.

We conducted a multicenter study to evaluate the performance of the FluoroType Mycobacteria VER 1.0 and the MBT Mycobacteria assays, as compared to the GenoType line probe assays, for the identification of NTM species isolated from clinical specimens. We also assessed the utility of these assays in clinical microbiology laboratories.

## MATERIALS AND METHODS

### Clinical isolates

A total of 119 clinical NTM isolates, including 85 slow-growing mycobacteria (SGM) and 34 rapid-growing mycobacteria (RGM), representing 33 species, were analyzed. This collection comprised (i) 76 isolates prospectively collected between January and December 2022 at the University Hospital Center of Bordeaux, France, and (ii) 43 isolates retrospectively collected between 2015 and 2022 from the University Hospital Centers of Bordeaux (*n* = 19), Nîmes (*n* = 17), and Rennes (*n* = 7), France (Table S1).

### Reference for comparison

NTM identification was performed on clinical isolates grown in liquid media (Mycobacteria Growth Indicator Tube, MGIT) or solid media (Lowenstein-Jensen, Coletsos). Initial identification was attempted using the GenoType Mycobacterium CM assay. For specimens with inconclusive results from the CM assay, the GenoType Mycobacterium AS assay was subsequently performed. Differentiation of species within the *M. avium* complex and subspecies within the *M. abscessus* complex was achieved using the GenoType NTM-DR assay. When species-level identification could not be achieved with these assays, 16S rRNA gene sequencing with subsequent phylogenetic analysis of 1,485 bp-amplicons was conducted (14).

### Clinical isolate preparation

All clinical mycobacterial isolates were subcultured in MGIT prior to analysis to obtain fresh cultures. For some isolates, subcultures were also performed on Lowenstein-Jensen or Coletsos medium to compare assay performance across different culture media. Tests were conducted when a positive signal was detected on liquid media by the BACTEC 460 TB radiometric system (Becton Dickinson Diagnostic Instruments, Sparks, Md.), or sufficient biomass was obtained in solid media (at least three colonies).

### FluoroType Mycobacteria assay

The FluoroType Mycobacteria assay was conducted according to the manufacturer’s instructions. Mycobacterial DNA was extracted from cultured isolates using the FluoroLyse kit (Hain LifeScience). An exogenous internal control DNA, provided in the kit, was added to each sample prior to DNA extraction. Amplification was performed on the FluoroCycler XT instrument (Hain Lifescience). Data were analyzed using the FluoroSoftware XT IVD available on the FluoroCycler XT instrument. Internal positive and negative controls were systematically included in each run. The assay was repeated for isolates with either “invalid” results or “no mycobacterial DNA detected” using a newly prepared extract from the same subculture. Repeat testing was also performed on cultures with discordant results using the same extract. If the species identification was still different from that expected, 16S rRNA gene sequencing was performed (14).

### MBT Mycobacteria assay

Mycobacterial proteins were extracted from cultured isolates according to the MBT Mycobacteria kit IVD protocol. All samples were spotted in triplicate onto the MALDI-TOF MS plate. Spectra acquisition was performed using the Bruker SMART MALDI-TOF MS instrument (Bruker Daltonics, Bremen, Germany), and spectra analysis was performed with the IVD MBT Mycobacteria Library v6.0 (Bruker Daltonics). Results were interpreted according to the manufacturer’s instructions: scores of ≥1.80 were considered “high-confidence identification,” scores between 1.79 and 1.60 were considered “low-confidence identification,” and scores ≤1.59 indicated “no organism identification possible.” For identification score analysis, the highest score for each spot was considered.

Analysis was repeated for isolates with either “no peak found” results or low scores (≤1.59). These isolates were subcultured, new protein extract was prepared, spotted in triplicate, and the analysis was repeated. Repeat testing was also performed on samples with discordant results. If the species identification was still different from that expected, 16S rRNA gene sequencing was performed (14).

### Data analysis

The performance of each assay was determined by calculating the overall percentage agreement values compared to the reference method. Statistical analyses were performed using the BiostaTGV website (https://marne.u707.jussieu.fr/biostatgv/). Dot plots were generated using R software and the ggplot2 package.

## RESULTS

The main characteristics of the FluoroType Mycobacteria and MBT Mycobacteria assays are compared to those of the GenoType line probe assays in Table 1. All kits were intended for *in vitro* diagnostics.

**TABLE 1:** Main characteristics of the three commercial assays^*d*^

Parameters	GenoType CM VER 2.0 GenoType AS VER 1.0 GenoType NTM-DR (Hain Lifesciences)	FluoroType Mycobacteria VER 1.0 (Hain Lifesciences)	MBT Mycobacteria (Bruker)
Technology	Line probe assay, PCR, and reverse hybridization DNA STRIP	Real-time PCR, melting curve analysis, and Liquid array technology	MALDI-TOF mass spectrometry
No. of species detected by the kit(s)	31 NTM species (+mutations associated with macrolide and aminoglycoside resistance)	32 NTM	182 Mycobacteria species
Extraction kit	GenoLyse	FluoroLyse	MBT Mycobacteria kit
Type of sample	Positive culture	Positive culture	Positive culture
Input volume	1 mL (liquid media) 1 µL inoculation loop (solid media)	500 µL (liquid media) 1 µL inoculation loop (solid media)	1.2 mL (liquid media) 3 µL inoculation loop (solid media)
IC^*a*^	Yes	Yes^*b*^	Yes
Manual steps	DNA extraction Reverse hybridization	DNA extraction	Protein extraction
Hands on time	~3 h 30 min	~45 min	~45 min
Automation steps	DNA amplification	DNA amplification and interpretation	Spectra acquisition and analysis
Test turnaround time	~5 h including 1 h 30 min of DNA amplification	~2 h 30 min including 1 h 45 min of DNA amplification	~1 h
No. of reactions/run	Up to 12 samples (can run distinct molecular tests)	Up to 94 samples (can run distinct molecular tests)	Up to 94 samples
Instrument	PCR system TwinCubator (Hain Lifescience GmbH)	FluoroCycler XT (Hain Lifesciences) Semi-Automated Sheet Heat Sealer (Azenta Life Sciences)	Bruker mass spectrometer (Bruker daltonics)
Consumable(s) not included	None	FrameStar 96 Well Skirted PCR Plate Sealing film Clear Weld Seal Mark II	MALDI MS plate Acetonitrile 70% formic acid
Data analysis software	NA	FluoroSoftware XT IVD	MALDI IVD Mycobacteria Library v6.0
List price/reaction (€), including taxes^*c*^	25.2 €/reaction	35.9 €/sample	9.5 €/sample

^*a*^ GenoType CM, AS, and FluoroType Mycobacteria VER1.0 kits use an exogenous internal control which is added in each sample prior DNA extraction. For the MBT Mycobacteria kit, an internal quality control (Bacterial Test Standard, BTS, Bruker Daltonics) is systematically spotted onto each plate.

^*b*^ Positive and negative controls must be used in each run with the FluoroType Mycobacteria assay.

^*c*^ Including DNA/protein extraction and consumables.

^*d*^ IC, internal control; NA, not applicable.

### FluoroType Mycobacteria assay

The percentage agreement between the FluoroType Mycobacteria assay and the reference method was 89.9% (107/119; Table 2). This agreement was 88.2% (75/85) for SGM and 94.1% (32/34) for RGM (*P* = 0.51).

**TABLE 2:** Results obtained with the FluoroType Mycobacteria VER 1.0 and the MBT Mycobacteria assays for NTM identification

NTM species (no. of isolates tested)	Identification method used routinely (reference method)	Expected identification with the FluoroType Mycobacteria VER 1.0 assay	FluoroType Mycobacterium results (expected identification/total)			Expected identification with the MBT Mycobacteria assay	MBT Mycobacterium results (expected identification/total)		
			Solid media	Liquid media	% agreement		Solid media	Liquid media	% agreement
*M. avium* (*n* = 20)	GenoType CM	*M. avium*	9/9	11/11	100	*M. avium*	9/9	10/11	95
*M. chelonae* (*n* = 7)		*M. chelonae*	4/4	3/3	100	*M. chelonae*	3/4	2/3	71.4
*M. fortuitum* group (*n* = 7)		*M. fortuitum*	4/4	3/3	100	*M. fortuitum* complex	3/4	1/3	57.1
*M. gordonae* (*n* = 9)		*M. gordonae*	3/3	3/6	66.7	*M. gordonae*	3/3	5/6	88.9
*M. interjectum* (*n* = 1)		*M. interjectum*	–^*c*^	0/1	0	*M. interjectum*	–	0/1	0
*M. kansasii* (*n* = 6)		*M. kansasii*	3/3	3/3	100	*M. kansasii*	3/3	3/3	100
*M. malmoense* (*n* = 1)		*M. malmoense*	–	1/1	100	*M. malmoense*	–	1/1	100
*M. marinum* (*n* = 4)		*M. marinum*	1/1	3/3	100	*M. marinum*	1/1	3/3	100
*M. scrofulaceum* (*n* = 1)		*M. scrofulaceum*	–	1/1	100	*M. scrofulaceum*	–	1/1	100
*M. xenopi* (*n* = 8)		*M. xenopi*	2/2	4/6	75	*M. xenopi*	2/2	3/6	62.5
*M. abscessus* ssp. *abscessus* (*n* = 8)	GenoType CM + NTM-DR	*M. abscessus* ssp. *abscessus*	1/1	7/7	100	*M. abscessus*	0/1	5/7	62.5
*M. abscessus* ssp. *massiliense* (*n* = 3)		*M. abscessus* ssp. *massiliense*	–	3/3	100	*M. abscessus*	–	3/3	100
*M. chimaera* (*n* = 5)		*M. chimaera*	1/1	4/4	100	*M. intracellulare_chimaera*	1/1	4/4	100
*M. intracellulare* (*n* = 17)		*M. intracellulare*	3/3	11/14	82.4	*M. intracellulare_chimaera*	3/3	13/14	94.1
*M. asiaticum* (*n* = 1)	GenoType CM + AS	*M. asiaticum*	–	1/1	100	*M. asiaticum*	–	0/1	0
*M. genavense* (*n* = 2)		*M. genavense*	–	1/2	50	*M. genavense*	–	0/2	0
*M. haemophilum* (*n* = 1)		*M. haemophilum*	–	1/1	100	*M. haemophilum*	–	0/1	0
*M. lentiflavum* (*n* = 2)		*M. lentiflavum*	–	2/2	100	*M. lentiflavum*	–	2/2	100
*M. mucogenicum* (*n* = 1)		*M. mucogenicum*	–	1/1	100	*M. mucogenicum_phocaicum*_ group	–	1/1	100
*M. shimoidei* (*n* = 1)		*M. shimoidei*	–	1/1	100	*M. shimoidei*	–	1/1	100
*M. simiae* (*n* = 1)		*M. simiae*	–	1/1	100	*M. simiae*	–	1/1	100
*M. szulgai* (*n* = 1)		*M. szulgai*	–	1/1	100	*M. szulgai*	–	0/1	0
*M. arupense*^*a*^ (*n* = 1)	GenoType CM + AS then 16S rRNA gene sequencing	*Mycobacterium* sp.	–	1/1	100	*M. arupense*	–	0/1	0
*M. bohemicum*^*b*^ (*n* = 1)		*Mycobacterium* sp.	–	1/1	100	*M. bohemicum*	–	0/1	0
*M. canariasense*^*b*^ (*n* = 1)		*Mycobacterium* sp.	–	1/1	100	*M. canariasense*	–	1/1	100
*M. elephantis*^*b*^ (*n* = 1)		*Mycobacterium* sp.	–	1/1	100	*M. elephantis*	–	1/1	100
*M. europaeum*^*b*^ (*n* = 2)		*Mycobacterium* sp.	1/1	1/1	100	*M. europaeum*	0/1	0/1	0
*M. hassiacum*^*b*^ (*n* = 1)		*Mycobacterium* sp.	–	1/1	100	*M. hassiacum*	–	1/1	100
*M. heraklionense*^*b*^ (*n* = 1)		*Mycobacterium* sp.	–	1/1	100	*M. heraklionense*	–	0/1	0
*M. mageritense^a^* (*n* = 1)		*Mycobacterium* sp.	–	0/1	0	*M. mageritense*	–	1/1	100
*M. novocastrense*^*b*^ (*n* = 1)		*Mycobacterium* sp.	–	1/1	100	*M. novocastrense*	–	1/1	100
*M. peregrinum* (*n* = 1)		*M. peregrinum*	–	1/1	100	*M. peregrinum*	–	1/1	100
*M. phocaicum*^*b*^ (*n* = 1)		*Mycobacterium* sp.	–	0/1	0	*M. mucogenicum_phocaicum*_ group	–	0/1	0
Total			32/32	75/87	89.9		28/32	65/87	78.15

^*a*^ Isolates evaluated by the manufacturer and identified as *Mycobacterium* sp. by the FluoroType Mycobacteria assay.

^*b*^ Species not evaluated by the FluoroType Mycobacteria assay.

^*c*^ –, no isolate was tested.

In one case, the FluoroType Mycobacteria assay demonstrated higher identification accuracy compared to the GenoType CM assay, correctly identifying *Mycobacterium peregrinum* at species level instead of as *Mycobacterium fortuitum* group. This identification was confirmed by 16S rRNA gene sequencing (Table 3).

**TABLE 3:** Characteristics of discrepant results^*d*^

Mycobacterial species	Culture media	Reference methods	Result obtained with the FluoroType Mycobacteria VER 1.0 assay	Result obtained with the MBT Mycobacteria assay
*M. abscessus* ssp. *abscessus*	Solid	GenoType CM/NTM-DR	*M. abscessus* ssp. *abscessus*	***Mycobacterium* sp.**
*M. abscessus* ssp. *abscessus*	Liquid		*M. abscessus* ssp. *abscessus*	***Mycobacterium* sp.**
*M. abscessus* ssp. *abscessus*	Liquid		*M. abscessus* ssp. *abscessus*	***Mycobacterium* sp.**
*M. arupense*	Liquid	GenoType CM/AS, 16S rRNA gene sequencing	*Mycobacterium* sp.^*b*^	***Mycobacterium* sp.**
*M. asiaticum*	Liquid	GenoType CM/AS	*M. asiaticum*	***Mycobacterium* sp.**
*M. avium*	Liquid	GenoType CM	*M. avium*	**NPF**
*M. bohemicum*	Liquid	GenoType CM/AS, 16S rRNA gene sequencing	*Mycobacterium* sp.^*b*^	***Mycobacterium* sp.**
*M. chelonae*	Liquid	GenoType CM	*M. chelonae*	**NPF**
*M. chelonae*	Solid		*M. chelonae*	***Mycobacterium* sp.**
*M. europaeum*	Solid	GenoType CM/AS, 16S rRNA gene sequencing	*Mycobacterium* sp.^*b*^	***Mycobacterium* sp.**
*M. europaeum*	Liquid		*Mycobacterium* sp.^*b*^	***M. chimaera_intracellulare_*group**
*M. fortuitum* group	Liquid	GenoType CM	*M. peregrinum^a^*	**NPF**
*M. fortuitum* group	Liquid		*M. fortuitum*	**NPF**
*M. fortuitum* group	Solid		*M. fortuitum*	***Mycobacterium* sp.**
*M. genavense*	Liquid	GenoType CM/AS	***Mycobacterium* sp.**	**NPF**
*M. genavense*	Liquid	GenoType CM/AS, 16S rRNA gene sequencing	*M. genavense*	**NPF**
*M. gordonae*	Liquid	GenoType CM	***Mycobacterium* sp.**	**NPF**
*M. gordonae*	Liquid		*Mycobacterium* sp.	*M. paragordonae^c^*
*M. gordonae*	Liquid		*Mycobacterium* sp.	*M. paragordonae^c^*
*M. haemophilum*	Liquid	GenoType CM/AS	*M. haemophilum*	**NPF**
*M. heraklionense*	Liquid	GenoType CM/AS, 16S rRNA gene sequencing	*Mycobacterium* sp.^*b*^	***Mycobacterium* sp.**
*M. interjectum*	Liquid	GenoType CM/AS, 16S rRNA gene sequencing	***Mycobacterium* sp.**	***Mycobacterium* sp.**
*M. intracellulare*	Liquid	GenoType CM/NTM-DR	*M. intracellulare*	***Mycobacterium* sp.**
*M. intracellulare*	Liquid		*Mycobacterium* sp.	*M. colombiense^c^*
*M. intracellulare*	Liquid		***Mycobacterium* sp.**	*M. chimaera_intracellulare_*group
*M. intracellulare*	Liquid		***Mycobacterium* sp.**	*M. chimaera_intracellulare_*group
*M. kansasii*	Solid	GenoType CM	*M. kansasii*	*M. gastri^c^*
*M. mageritense*	Liquid	GenoType CM/AS, 16S rRNA gene sequencing	***M. smegmatis***	*M. mageritense*
*M. malmoense*	Liquid	GenoType CM	*M. malmoense*	*M. palustre^c^*
*M. phocaicum*	Liquid	GenoType CM/AS, 16S rRNA gene sequencing	***M. mucogenicum***	**NPF**
*M. szulgai*	Liquid	GenoType CM/AS	*M. szulgai*	***Mycobacterium* sp.**
*M. xenopi*	Liquid	GenoType CM	***Mycobacterium* sp.**	***Mycobacterium* sp.**
*M. xenopi*	Liquid		**No mycobacterial DNA detected**	**NPF**
*M. xenopi*	Liquid		*M. xenopi*	***Mycobacterium* sp.**

^*a*^ Member of the *M. fortuitum* group.

^*b*^ Results considered as concordant because the species tested are not detected by the FluoroType Mycobacteria assay.

^*c*^ Results considered as concordant because 16S rRNA gene sequencing confirmed the species identified with the MBT Mycobacteria assay.

^*d*^ Discordant/discrepant results compared to our reference method are indicated in bold. NPF, no peak found.

Seven species not included in the panel of the FluoroType Mycobacteria assay were correctly identified at the genus level in our data set: *Mycobacterium bohemicum*, *Mycobacterium europaeum*, *Mycobacterium heraklionense*, *Mycobacterium canariense*, *Mycobacterium elephantis*, *Mycobacterium hassiacum*, and *Mycobacterium novocastrense* (Table 2).

The assay was repeated for 16 (13.4%) isolates with unsuccessful results on initial testing. Despite repeat testing, identification remained inconclusive for 12 isolates, corresponding to a failure rate of 10.1%. Of these, nine were identified only to the genus level (*Mycobacterium xenopi* [*n* = 1], *Mycobacterium intracellulare* [*n* = 3], *Mycobacterium genavense* [*n* = 1], *Mycobacterium interjectum* [*n* = 1], and *Mycobacterium gordonae* [*n* = 3]), while two were misidentified (*Mycobacterium smegmatis* instead of *Mycobacterium mageritense*, and *Mycobacterium mucogenicum* instead of *Mycobacterium phocaicum*), and one *M. xenopi* isolate yielded “no mycobacterial DNA” result (Table 3). Notably, all discordant results originated from mycobacteria cultured in liquid media (12/87). Except for *M. mucogenicum*, the specificity per organism of the FluoroType Mycobacteria assay was 100% (Table S2).

Among the most frequently encountered species in clinical settings, low concordance was observed for *M. intracellulare* (82.4%, 14/17), *M. gordonae* (66.7%, 6/9), and *M. xenopi* (75%, 6/8; Table 2).

### MBT Mycobacteria assay

The percentage agreement between the MBT Mycobacteria assay and the reference method was 78.1% (93/119) (Table 2). More SGM (81.2%, 69/85) was correctly identified than RGM (73.5%, 25/34; *P* = 0.45). Overall, more isolates cultured on solid media (87.5%, 28/32) were correctly identified than those cultured in liquid media (74.7%, 65/87; *P* = 0.21; Table 2).

Misidentifications occurred for closely related species: *Mycobacterium gastri* instead of *Mycobacterium kansasii, M. paragordonae* instead of *M. gordonae* (*n* = 2), *Mycobacterium palustre* instead of *Mycobacterium malmoense*, and *Mycobacterium colombiense* instead of *M. intracellulare*. However, these species were not detected by the line probe assays, and further 16S rRNA gene sequencing confirmed the MALDI-TOF identification, indicating that the results were ultimately concordant (Table 3).

On initial testing, 29 isolates (8 RGM and 21 SGM) generated “no peak found” results on triplicate spots. Repeat testing did not resolve the issue for 10 isolates, provided genus-level identification for four isolates, and resulted in one misidentification (*M. intracellulare*/*Mycobacterium chimaera* instead of *M. europaeum*; Table 3). All of these isolates were cultured in liquid media. The specificity per organism was 100%, except for *M. intracellulare*/*chimaera* (99%; Table S2).

Additionally, 18 other isolates could not be identified to the species level (score of <1.59) on initial testing. Spotting with a new protein extract resulted in the same issue for 11 isolates (Table 3). Overall, the failure rate of the MBT Mycobacteria assay was 39.5% (47/119) on initial testing and was reduced to 18.5% (22/119) on repeat testing.

**Fig 1:**
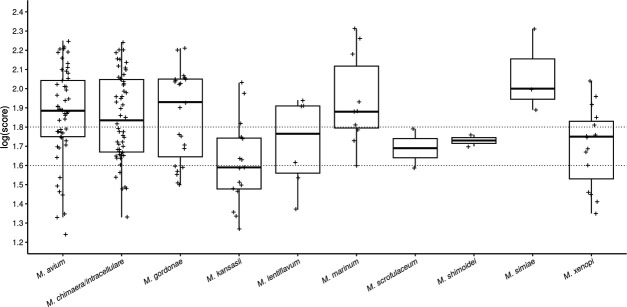
Log(score) distribution for slow-growing mycobacterial species. All log(score) values obtained for all isolates tested in triplicate are represented. No peak was found for some replicates.

Among SGM, *M. kansasii* had the lowest median log(score) with a median of 1.59 (range: 1.11–2.03; Fig. 1). Five species were identified with a log(score) >1.8, with *Mycobacterium simiae* yielding the highest log(score). The log(score) was significantly higher when the assay was performed on isolates cultured on solid rather than liquid media for *M. gordonae*, *M. avium*, *M. kansasii, Mycobacterium marinum*, and *M. xenopi* (*P* < 0.05; Fig. S1).

**Fig 2:**
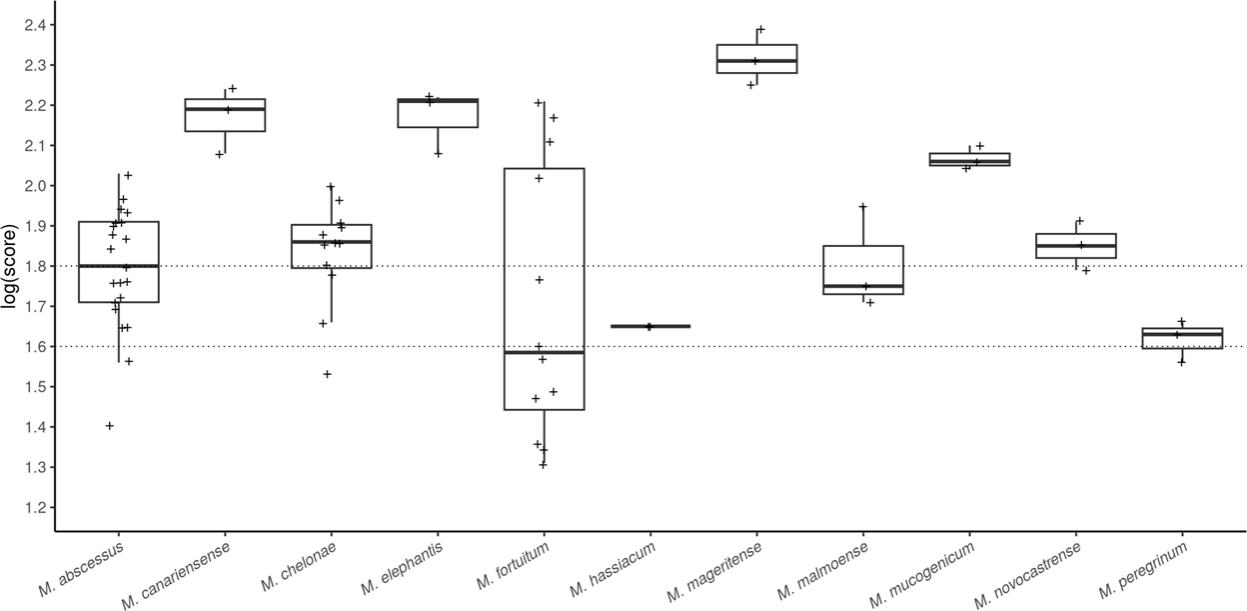
log(score) distribution for for rapid-growing mycobacterial species. All log(score) values obtained for all isolates tested in triplicate are represented. No peak was found for some replicates.

Regarding RGM, *M. fortuitum* obtained the lowest log(score) (median 1.59, range:1.31–2.21), particularly for isolates cultured on solid media (Fig. 2; Fig. S1). *M. mageritense* and *M. elephantis* had higher log(scores).

## DISCUSSION

This study demonstrated that the FluoroType Mycobacteria VER 1.0 and the MBT Mycobacteria assays were useful tools for NTM identification.

The FluoroType Mycobacteria assay successfully identified 89.9% (107/119) of the NTM isolates tested. This rate was higher than that reported in two recent studies, where 85.5% (126/148) and 87.8% (79/90) of the NTM species were correctly identified (15, 16). The identification accuracy for RGM was higher (94.1%) than that for SGM at 88.2%, consistent with previous report (15). The observed discordance between *M. smegmatis* and *M. mageritense* in our and previous study could be because both species may have the same fluorescence signature (15). Similarly, *M. phocaicum* and *M. mucogenicum* belong to the same phylogenetic group, which could explain the misidentification in our data set (17). None NTM misidentifications were observed with isolates cultured on solid media compared to liquid media. Such difference was not reported by Luukinen et al. (15). Despite good overall performance, this assay showed weakness in identifying *M. intracellulare*, *M. xenopi*, and *M. gordonae*, three commonly encountered species in clinical microbiology laboratories. Among the 17 *M*. *intracellulare* isolates tested, only 14 (82.4%) were correctly identified. In other studies, successful identification for this species ranged from 42% (5/12) to 100% (5/5) (15, 16). For *M. xenopi*, only 75% (6/8) of the isolates were correctly identified in our data set, which was a lower rate than that previously reported, although fewer isolates were tested in those studies (15, 16). *M. gordonae* was identified as *Mycobacterium* sp in one-third of the cases in our study, compared to 50% and 40% of the cases in two previous studies (15, 16).

The MBT Mycobacteria assay correctly identified SGM species in 81.2% (69/85) of the cases. Discrepancies may be explained by the presence of less frequently encountered species in our data set that are underrepresented in the Mycobacteria Library v6.0, such as *M. heraklionense*, *M. genavense*, *Mycobacterium haemophilum*, and *M. europaeum*. The rate of successful RGM identification was lower (73.5%, 25/34), contrasting with previous studies showing higher accuracy of MALDI-TOF identification for RGM compared to SGM (13, 18). This may be due to the different intrinsic characteristics of the clinical isolates included in this study compared to those in the database, resulting in low log(score) values (13). Clinical isolate belonging to the same species could have different proteome according to their geographical origin, for instance (19). An alternative hypothesis is the difficulty of assessing biomass in liquid media, in which most of the RGM isolates were cultured, contributing to the absence of peaks in 8.4% (10/119) of isolates. Therefore, it is recommended to analyze mycobacterial cultures a few days after the positive detection signal and to spot protein extracts in triplicate to overcome differences in spectral acquisition (11, 12, 19, 20). Prior to analysis, the presence of bacilli was verified by Ziehl-Neelsen staining. Additionally, culture media could impact NTM identification (19, 21). In our data set, not all isolates were evaluated in both liquid and solid media. However, identification results were higher for isolates cultured on solid media than on liquid media. Recent studies showed better performance with the Middlebrook 7H11 medium than with MGIT medium (20, 22). Indeed, spectral variations with the presence or absence of some peaks can occur depending on the medium used and owing to the presence of antimicrobials and additional growth in liquid cultures (19). Culture on agar medium has the advantage of isolating different colonies, but growth on this medium can be challenging for some NTM, and diagnosis may be delayed. For inconclusive results, we showed that repeat testing with new protein extraction identified an additional 53.2% (25/47) of isolates. This suggests that variability in protein extraction and spectra acquisition can occur despite the use of quality control in each experiment, laser calibration, and standardization of protein extraction method (20). Despite the improvement of the recently marketed extraction method (20), an additional ultrasound step may improve the success rate for rough strains by dissociating the aggregates (19). Most species were identified with a log(score) >1.8, but our results suggested that species-specific cut-off values should be considered for some NTM (18). A limitation of the FluoroType Mycobacteria and GenoType assays compared to MALDI-TOF MS is their static reference database. In contrast, the MBT Mycobacteria library is continuously updated, enabling the identification of a broad spectrum of NTM, including less frequently encountered and closely related species (13, 18, 23). However, in contrast to the FluoroType Mycobacteria and the GenoType tests, the MBT Mycobacteria assay could not differentiate between *M. intracellulare* and *M. chimaera* or the *M. abscessus* subspecies. Nevertheless, an additional sub-typing module with further spectral interpretation algorithms or machine learning methodologies can be used for this purpose (19, 24, 25). GenoType assays require a two-step process for accurate identification of these species and subspecies.

A strength of this study was the exclusive use of clinical isolates to evaluate the assays. These strains may exhibit distinct intrinsic characteristics compared to culture collection strains (19). In order to be representative and useful in clinical practice, most isolates were collected prospectively, reflecting our local epidemiology. The GenoType assays identified 94.7% (72/76) of them at the species level. We also tested eight species that have never been tested with the FluoroType Mycobacteria assay. This study also had some limitations. For less frequently encountered NTM, we tested single isolates, which could have led to misidentification at the genus level. Although our reference method (GenoType assays, followed by 16S rRNA gene sequencing if no identification was obtained) is commonly used among European mycobacterial laboratories (10), identification of closely related species can be potentially biased. For instance, two isolates identified as *M. gordonae* by the GenoType CM assay were identified as *M. paragordonae* by MALDI-TOF MS, which had no clinical impact as both species are generally non-pathogenic. Another isolate identified as *M. intracellulare* by our reference method was identified as *M. colombiense* by MALDI-TOF MS, both species belonging to the *M. avium* complex. *M. colombiense* is known to cause disseminated infection in both immunocompromised and immunocompetent individuals (26). One isolate identified as *M. kansasii* by the routinely used GenoType CM and the FluoroType assays was identified as *M. gastri* by MALDI-TOF MS. While these two species are closely related species, their pathogenicity varies significantly, with *M. kansasii* being more pathogenic than *M. gastri* (27). Microbiologist must be aware of the limits of the method used for the identification of NTM species and may need to use additional tests to achieve a more accurate identification, according to the clinical context. Another limitation of this study was the absence of *M. abscessus* subsp. *bolletii* isolates in our data set due to its very low frequency in our local epidemiology. We were therefore unable to assess the performances of the FluoroType for the identification of this subspecies. Finally, we did not test cultures with multiple species. Previous studies have reported that the FluoroType Mycobacteria is unable to identify multiple species in a single sample (15). Mixed infections can be suspected with GenoType assays when multiple stripes, not specific to a single species, are observed.

In conclusion, the FluoroType Mycobacteria VER 1.0 and the MBT Mycobacteria assays were found to be useful tools, with reduced handling time for NTM identification from positive cultures, compared to GenoType assays. However, the identification of NTM grown in liquid media showed moderately successful performance for both systems. Their implementation in routine laboratory practice should consider their performance characteristics and limitations within a clinical setting. The FluoroType Mycobacteria identifies the same species as the GenoType assays in a single run, with improved workflow, which may be of interest for medium-to-large laboratories. However, in our setting, the cost of reagent is the main limitation to its routine use. The MBT Mycobacteria assay offers a wider range of NTM species identification, but its reliability as a first-line method for identification from liquid media still needs to be optimized. In our routine procedures, the MBT Mycobacteria assay will be used as a second-line method from isolates subcultured on solid media.

## SUPPLEMENTAL MATERIAL

Figure S1 jcm.01206-24-s0001.tif Log(score) obtained for mycobacterial species from liquid media and solid media.

Table S1 jcm.01206-24-s0002.xlsx Characteristics of mycobacterial clinical isolates analyzed in this study and corresponding results obtained with the two commercial assays.

Table S2 jcm.01206-24-s0003.xlsx Specificity analysis for the FluoroType Mycobacteria VER 1.0 assay and the MBT Mycobacteria assay.

ASM does not own the copyrights to Supplemental Material that may be linked to, or accessed through, an article. The authors have granted ASM a non-exclusive, world-wide license to publish the Supplemental Material files. Please contact the corresponding author directly for reuse.
