# Supplementary figures and images for: Comparison of the molecular FluoroType Mycobacteria VER 1.0 and the Maldi BioTyper Mycobacteria assays for the identification of non-tuberculous mycobacteria
Source: J Clin Microbiol. 2024 Dec 11;63(1):e01206-24. doi: 10.1128/jcm.01206-24 (PMC11784439; doi:10.1128/jcm.01206-24)

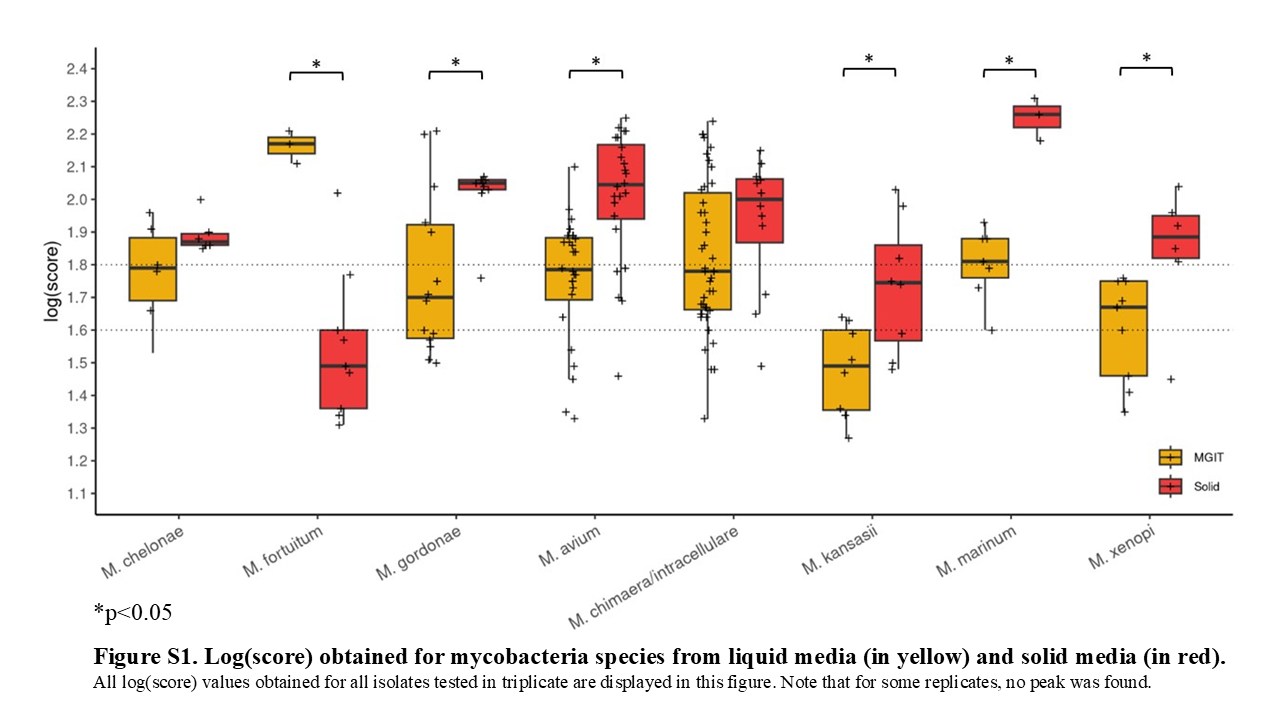

Supplement: Figure S1 — Log(score) obtained for mycobacterial species from liquid media and solid media. [file jcm.01206-24-s0001.tif]
